# Environmental Regulation, Financial Pressure and Industrial Ecological Efficiency of Resource-Based Cities in China: Spatiotemporal Characteristics and Impact Mechanism

**DOI:** 10.3390/ijerph191711079

**Published:** 2022-09-04

**Authors:** Yiming Hou, Guanwen Yin, Yanbin Chen

**Affiliations:** College of Geography and Environment, Shandong Normal University, No. 1 Daxue Road, University Science Park, Changqing District, Jinan 250358, China

**Keywords:** industrial ecological efficiency, environmental regulation, financial pressure, influential mechanism, resource-based cities

## Abstract

A resource-based city is a type of city characterized by the exploitation and processing of natural resources as the leading industry in the region. Such cities provide essential resources for China’s economic development and support long-term rapid economic growth. However, resource-based cities (RBCs) face challenges, including resource depletion, economic recession, environmental pollution, and ecological damage, to which not enough attention has been paid. In the context of China’s increased focus on environmental protection and the economy, improving industrial ecological efficiency of RBCs has become ever more important. In the present study, the Super-SBM model was used to measure the industrial ecological efficiency of 114 RBCs in China from 2003 to 2016. The results show that during the study period, the industrial ecological efficiency of RBCs in China improved significantly, particularly in the central and western regions. The results from a Tobit model show that appropriate environmental regulation and financial pressure have a positive impact on the industrial ecological efficiency of RBCs. However, when faced with the dual pressures of environmental regulation and financial difficulty, improvement in industrial ecological efficiency was inhibited. The impact of environmental regulation and financial pressure on industrial ecological efficiency of cities in different regions and development stages and with different resource types shows heterogeneity. In accordance with the study findings, differentiated measures and suggestions are proposed to improve the industrial ecological efficiency of RBCs.

## 1. Introduction

### 1.1. Necessity of Study

China’s rapid industrialization since the implementation of the reform and opening-up policy has greatly promoted economic development. However, industrial development is often accompanied by several problems, including high energy consumption, high pollution, and low efficiency. The resulting ecological and environmental problems have become a major obstacle to China’s sustainable development [1]. In 2015, the Chinese government issued the “Overall Plan for the Reform of Ecological Civilization System” to gradually improve the legal and market system for ecological and environmental protection. Specifically, environmental regulation has played a positive role in promoting economic growth and improving the ecological environment. At the same time, a series of institutional reforms in finance (e.g., the abolition of agricultural tax and the replacement of business tax with value-added tax) has widened the gap between local governments’ fiscal revenue and their expenditure, leading to heavy pressure on ecological and environmental protection [2]. In the context of increasing environmental regulation and financial pressure, local governments need to coordinate the relationship between economic growth, resource utilization, and environmental protection, thereby improving industrial ecological efficiency (IEE).

IEE not only represents the environmental impacts of expansion of industrial production, but also industrial productivity under environmental constraints. This is a common indicator for evaluating the relationship between industrial development and the ecological environment. Recent studies on IEE have been carried out mainly from three aspects: (1) research scale: different studies are carried out at the country [3,4,5], region [6,7], city [8,9], or enterprise [10,11] level; (2) research method: common methods include data envelope analysis (DEA) [12,13,14], ecological footprint method [15,16], energy analysis [17,18,19], and material flow analysis [20,21,22] (DEA is the most widely used method since it evaluates the weight of the endogenous indices and has strong objectivity); and (3) influencing factors: econometric models are used to study the influences of economic development [23], industrial structure [24], energy structure [25], and foreign capital utilization [26] on IEE.

Environmental regulation protects the ecological environment via administrative orders, market regulations, and public participation. Regarding the impact of environmental regulation on ecological efficiency, many theoretical and empirical studies have been conducted based on different assumptions, research samples, analysis methods, and variable designs. Some studies found that environmental regulation effectively improved environmental quality [27], enhanced public health [28], and led to green innovation and industrial upgrading [29,30]. To a certain extent, environmental regulation promotes improved ecological efficiency. However, some studies point out that the rising cost brought about by environmental regulation cannot be ignored. For example, the implementation of environmental regulation can lead to transfer of environmental pollution across regions and form a “pollution paradise” that inhibits regional economic growth in the short term [31].

Financial pressure refers to the degree of fiscal deficit due to the difference between fiscal revenue and government expenditure; this reflects the fiscal gap of local governments [32]. Financial pressure is caused mainly by chronic imbalances among government responsibility, service demand, capital investment, and the ability of the government to attract revenue [33]. Since fiscal decentralization reform, China has entered a stage of centralized financial power and localized administrative power [34]. Due to their responsibility for infrastructure construction and improvement of people’s livelihood, local governments face great financial pressure [35]. A number of recent studies have focused on Chinese-style financial pressure from the perspectives of resource utilization [36], local governance reform [37], pollution control efficiency [38,39], and the quality of economic development [40]. These studies show that financial pressure changes local government strategy with respect to the economy and environment [41], which subsequently has a strong impact on IEE.

As the key energy and resource supply base for national economic development, resource-based cities (RBCs) provide essential resources for China’s economic development and support long-term rapid economic growth [42]. Due to long-term, high-intensity mining of resources, RBCs face severe economic depression and ecological environment damage, which significantly restricts improvement of IEE [13]. There is a lack of research on the IEE of RBCs. Accordingly, this study uses the Super-SBM model to measure the IEE of 114 RBCs in China, as well as its temporal and spatial evolution characteristics. Based on the economic region, development stage, and dominant resource type of RBCs, the impact of environmental regulation and financial pressure on IEE is comprehensively investigated. This study promotes the current research efforts in the following three aspects: First, the Super-SBM model allows comparison of IEE of all Decision-Making Units (DMUs); thus, this study presents a clear demonstration of the level and spatial distribution of IEE. Second, the IEE of RBCs with prominent contradictions between ecological environment and economic development is studied, enriching research in this area. Third, in the context of China’s economy entering a “new normal” by attaching greater importance to the ecological environment, the mechanism of influence of environmental and financial pressure on IEE is explored, and the conclusions provide a framework for the government to formulate scientific policies for the sustainable development of RBCs.

### 1.2. Purpose

The purpose of this study is to identify the impact of environmental regulation and financial pressure on the IEE of RBCs. The specific purposes are:

(1) To identify the impact of environmental regulation and financial pressure on IEE of RBCs.
(2) To identify the comprehensive impact mechanism of environmental regulation and financial pressure on IEE of RBCs.

## 2. Theoretical Analysis

The Chinese government is placing increasing emphasis on environmental protection and rigorous environmental governance [43]. However, environmental regulation is always a double-edged sword, which may limit economic development while protecting the environment [44,45]. In the short term, rigorous environmental regulation means capital and technological constraints, which will hinder economic development to a certain extent, but in the long run, rigorous environmental regulation may improve economic efficiency [46,47,48]. The existing literature shows that environmental regulation can promote industrial eco-efficiency mainly in three aspects: First, it provides intrinsic incentives to improve IEE [49]. Resource-based enterprises will choose to increase technological investment, thereby increasing industrial diversity and reducing resource dependence [30,50]. Second, environmental regulation limits the resource consumption and pollution emissions of economic entities through legal or economic measures, thereby improving environmental quality and ecological efficiency [51]. Third, as a government intervention measure, environmental regulation indirectly enhances consumers’ green consumption awareness and preferences [52], forcing companies to upgrade their technological processes and promoting IEE. A study of China’s manufacturing industry found that the impact of environmental regulation showed significant intra-industry heterogeneity. Environmental regulation had a significant positive impact on heavily polluting sub-industries and moderately polluting sub-industries, but an insignificant effect in lightly polluting sub-industries [53]. Therefore, combined with the realistic background of resource-based cities in China, Conjecture (1) is put forward: Environmental regulation will promote the improvement of IEE of RBCs.

In the process of economic and social transformation, Chinese local governments are faced with top-down performance appraisal pressure, bottom-up demand satisfaction pressure, and horizontal development competition pressure. Such pressure is transformed mainly into financial pressure on local governments. In areas with high financial pressure, in order to cope with the sharp fiscal conflicts caused by the imbalance between fiscal revenue and expenditure, the local government will reduce the environmental protection requirements for enterprises and take expanding tax sources, appropriately reducing fiscal expenditure as an important way to relieve the pressure, which will negatively affect IEE [54]. Specifically, on the one hand, the local governments facing financial pressure will put pressure on the tax authorities, which will increase the pressure on tax collection and management and make it more difficult for enterprises to finance. On the other hand, the lack of financial resources of local governments may weaken the allocation efficiency of resources and environment, thus restricting the level of regional pollution control and green production innovation [55]. Further, under huge financial pressure, local governments are more inclined toward regional financial stabilization, and will take various measures to expand tax revenue. As a result, local governments may allow extensive environmentally unfriendly production methods by relaxing environmental regulations, introducing enterprises with excessive production capacity and condoning high-energy-consuming and high-polluting enterprises to evade local environmental penalties and supervision [38,56]. As Stewart pointed out, the local government with certain decision-making power, considering that the utility of improving environmental quality is less than the utility brought by the inflow of capital and other factors into the local area, will prefer capital inflow rather than environmental governance [57]. This will eventually lead to high energy consumption and deterioration of the ecological environment, hindering improvement of IEE. Therefore, Conjecture (2) is proposed: financial pressure will inhibit the improvement of IEE of RBCs.

The industrial structure of RBCs is relatively simple, the utilization rate of clean technology and environmentally friendly energy in production is low, and it is challenging to take into account both environmental and economic benefits. When RBCs face the dual pressures of environmental regulation and financial difficulty at the same time, investment by enterprises and government in environmental protection technology declines. Thus, the positive impact of environmental regulation on IEE may be cancelled out by the negative effects of financial pressure. Therefore, Conjecture (3) is put forward: the dual pressure of environmental regulation and financial gap has a negative effect on the IEE of RBCs.

**Figure 1:**
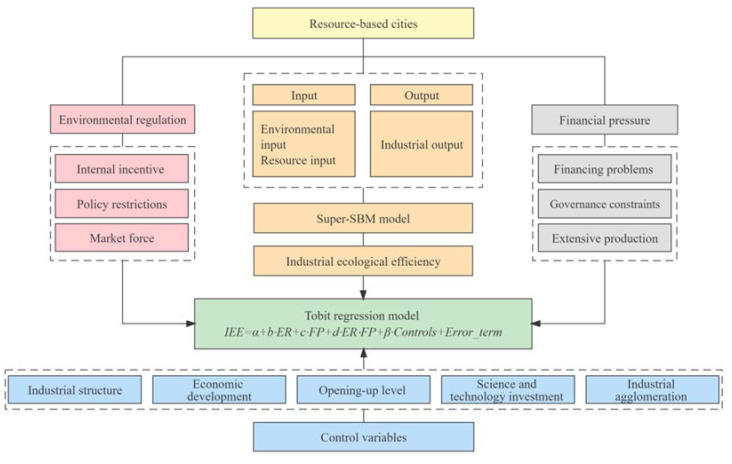
Research framework.

The research framework (Figure 1) of this paper is as follows:

## 3. Study Area

**Figure 2:**
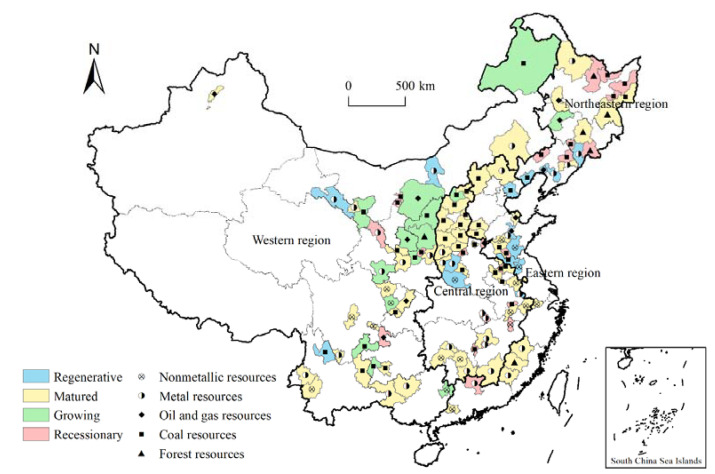
Spatial distribution of resource-based cities in China.

According to the National Sustainable Development Plan for RBCs in China (2013–2020), there are 262 resource-based administrative entities in the country, including 126 prefecture-level administrative regions, 62 county-level cities, 58 counties, and 16 municipal districts. Considering the comparability of the study areas and data availability, 114 prefecture-level RBCs were included as our research objects (Figure 2). From the perspective of economic location, RBCs are relatively concentrated in the central and western regions (32.5% and 33.3%, respectively), and a few are in the eastern and northeastern regions (17.5% and 16.7%, respectively). According to development stage, RBCs can be divided into four categories: growing, matured, recessionary, and regenerative. The development of resources in growing cities is in the growing stage, and the potential for resource security is high. In mature cities, resource development enters a stable stage, and large-scale expansion of resource-based industries continues. Recessionary cities tend to be depleted of resources, have stagnant economic development, and have prominent problems related to the environment and people’s livelihoods. Regenerative cities have passed the stage of heavy reliance on resources and are pioneering areas for economic transformation. According to the dominant resource type, RBCs can be divided into coal, metal, non-metal, forest, and oil and natural gas resource-based cities.

## 4. Methods and Materials

### 4.1. Methods

#### 4.1.1. The Super-SBM Model

DEA is a deterministic and non-parametric efficiency evaluation method proposed by Charnes et al. [58]. Compared with the stochastic or parametric efficiency measurement method, DEA does not need to set the specific form of production function and does not contain random error [59]. The relative efficiency of DMUs is evaluated based on the degree of deviation from the frontier of DEA, and it shows strong objectivity. Compared with traditional DEA models, the SBM model considers the influence of slack variables in the objective function, which solves the slack problem of input–output variables. The SBM model also takes into account the influence of undesired output on efficiency. However, the SBM model has not yet solved the problem of comparing multiple DMUs with an efficiency value of 1. To solve this problem, Tone proposed the Super-SBM model, which solves the problem of the DMU efficiency value being greater than 1, making it possible to compare multiple effective DMUs [60]. Therefore, the Super-SBM model is used to calculate the IEE of 114 RBCs in China from 2003 to 2016. The Super-SBM model is expressed as follows:

$$\rho =\min\frac{1+\frac{1}{m}\sum_{i=1}^{m}s_{i}^{-}/x_{ik}}{1-\frac{1}{s}\sum_{r=1}^{s}s_{r}^{+}/y_{rk}},$$

$$St.\;\{\begin{array}{c} \sum_{j=1,j\neq k}^{n}x_{ij}\lambda_{j}-s_{i}^{-}\leq x_{ij}\left(i=1,2,\cdot \cdot \cdot ,m\right),\\ \sum_{j=1,j\neq k}^{n}y_{rj}\lambda_{j}+s_{r}^{+}\geq y_{rk}\left(r=1,2,\cdot \cdot \cdot ,s\right),\\ \lambda_{i}\geq 0,j=1,2,\cdot \cdot \cdot ,n\left(j\neq k\right),s_{i}^{-}\geq 0,s_{r}^{+}\leq 0, \end{array},$$

where *x* and *y* are the input and output variables, respectively; *m* and *s* denote the number of input and output indicators of the DMU, respectively; *λ_j_* is the weight of each element in the reference set; $s_{i}^{-}$ and $s_{r}^{+}$ are the input and output slack variables, respectively; and *ρ* is the relative efficiency value.

#### 4.1.2. Tobit Regression Model

Since IEE is a restricted variable, parameter estimation using the traditional OLS model will cause significant bias and inconsistency [61]. The Tobit regression model uses the maximum likelihood method for parameter estimation and can obtain consistent and effective results that follow the normal distribution, thereby avoiding parameter inconsistency and bias. Therefore, a Tobit regression model with limited dependent variables is used to analyze the influencing factors of *IEE*. The model settings are as follows:

$$IEE_{it}=\alpha_{0}+\alpha_{1}ER_{it}+\alpha_{2}FP_{it}+\alpha_{3}ER_{it}\cdot FP_{it}+\beta_{it}Controls_{it}+\varepsilon_{i,t},$$

where *IEE_it_* is the explained variable of the IEE of city *i* in year *t*; *FP_it_* is the financial pressure of city *i* in year *t*; *ER_it_* is the environmental regulation intensity of city *i* in year *t*; *ER_it_*·*FP_it_* is an interaction term of environmental regulation and financial pressure; *Controls_it_* is a series of control variables; and *ε_it_* is a random disturbance term in the normal distribution.

### 4.2. Variable Selection

#### 4.2.1. Explained Variable

The IEE of RBCs is selected as the explained variable. The concept of ecological efficiency was proposed in the 1990s to measure the environmental impact of economic activity. This is calculated as the ratio of economic output to resource and environmental input [61]. Here, output refers to the value of products and services provided by the economy, and input refers to the resources and energy consumption of the economy and the associated environmental burden. The Super-SBM model reflects the impact of a certain resource input on the efficiency by examining the impact of the elastic change of the input resources on the construction of the random frontier of DEA. In this study, resource consumption or environmental pollution in the process of industrial production is referred to as environmental pressure, and the ratio of industrial output to the resulting environmental pressure is used to characterize IEE. IEE emphasizes maximum industrial economic output with the least resource consumption. Based on existing studies [24,26], and considering the representativeness, scientific value, and data availability of the indicators, this study selected industrial dust emissions, industrial wastewater discharge, and industrial SO_2_ emissions as environmental input indicators, and industrial electricity consumption as the resource input indicator. Total industrial output value was chosen as the output indicator. Descriptions of the indicators are shown in Table 1.

#### 4.2.2. Explanatory Variables

(1) Environmental regulation

Environmental regulation comprehensively reflects the intensity of a government’s environmental policies in environmental management, which can be characterized by emission reduction and pollution control effects. Considering the availability of necessary data and avoiding use of a single indicator, five indicators are included in this study: industrial SO_2_ removal efficiency, industrial dust removal rate, comprehensive utilization rate of industrial solid waste, treatment rate of domestic sewage, and harmless treatment rate of domestic waste. The entropy method was used to assign a weight to each indicator. According to the weights, the environmental regulation intensity of each resource-based city was obtained. The specific steps of the entropy method are described in Li et al. [62].

(2) Financial pressure

Financial pressure refers to the persistent gap between fiscal revenue and fiscal expenditure of a country or region. This study uses the ratio of fiscal gap to fiscal revenue to represent the level of financial pressure [63]. The equation is as follows:

$$FP_{i}=\frac{E_{i}-R_{i}}{R_{i}},$$

where *FP_i_* is the financial pressure index of resource-based city *i*, and *E_i_* and *R_i_* are fiscal expenditure and fiscal revenue of city *i*, respectively.

#### 4.2.3. Control Variables

To eliminate the influence of other factors on the explained variables, in accordance with previous studies [30,64], we selected five indicators as control variables: industrial structure, economic development level, opening-up level, science and technology investment, and industrial agglomeration.

(1) Industrial structure

Cutting overcapacity is an important way for RBCs to improve IEE, and industrial structure adjustment is the key to cutting overcapacity in RBCs. Optimization of the industrial structure means advanced and rationalized allocation of resources that is conducive to high-quality economic development [65]. In this study, the ratio of the output value of the secondary industry to regional GDP characterizes industrial structure.

(2) Economic development level

Economic development level is the basis and premise of improvement of IEE. Economic development promotes improved IEE by increasing industrial technological investment and green subsidies. This study uses per capita GDP to measure economic development level of RBCs, and the data are logarithmically processed.

(3) Opening-up level

The impact of the opening-up level on IEE is two-fold. First, an improvement in opening-up level can bring advanced technologies, equipment, and management expertise, which helps to improve IEE. Second, as China is rich in energy, labor, and other resources, the country specializes in production of labor- and resource-intensive products, which may increase environmental pressure for local governments [66]. We use the ratio of foreign investment to regional GDP to characterize the opening-up level of RBCs.

(4) Science and technology investment

As a key factor in improving innovation capability, science and technology investment plays an important role in the transformation and development of RBCs [67]. In the process of economic growth, it takes a certain amount of time to transform investment into scientific achievements that can drive improved IEE. We use the ratio of science and technology expenditure to financial expenditure to measure the level of science and technology investment of RBCs.

(5) Industrial agglomeration

The influence of industrial agglomeration on the environment is also two-fold. First, as the degree of industrial agglomeration increases, consumption of resources and energy increases, which in turn increases the degree of pollution. Second, pollution emissions in industrial agglomeration areas can be reduced through positive externalities such as environmental pollution control scale effects, environmental control technology spillovers, and industrial symbiosis [68]. Relative industrial density characterizes industrial agglomeration of RBCs; the equation is expressed as follows:

$$IA_{i}=\frac{Ind_{i}/Area_{i}}{\sum_{i=1}^{n}Ind_{i}/\sum_{i=1}^{n}Area_{i}},$$

where *IA_i_* represents the level of industrial agglomeration of resource-based city *i*; and *Ind_i_* and *Area_i_* are total industrial output value and administrative area of resource-based city *i*, respectively.

Descriptive characteristics of each variable are shown in Table 2.

The data for total industrial output value, industrial wastewater discharge, industrial SO_2_ emissions, industrial dust discharge, and other variables for the 114 RBCs included in this study are from the 2004–2017 China Urban Statistical Yearbook and China Urban Construction Statistical Yearbook, and individual missing data are compiled and supplemented from provincial statistical yearbooks.

## 5. Results and Discussion

### 5.1. Spatiotemporal Characteristics of IEE

**Figure 3:**
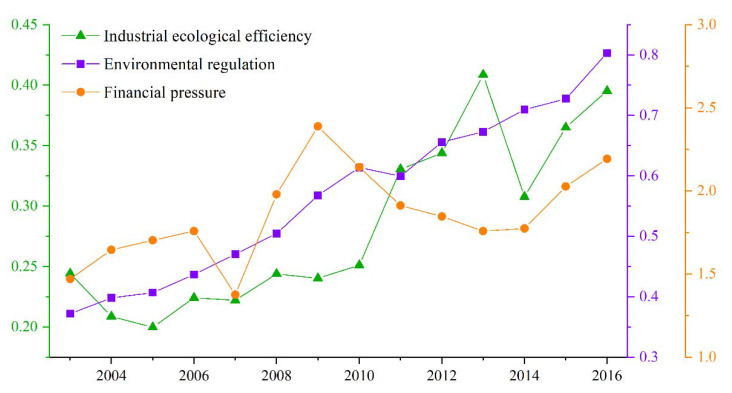
Temporal evolution of IEE, financial pressure, and environmental regulation.

Figure 3 shows the temporal trends of the annual averages of IEE, environmental regulation intensity, and financial pressure index for the 114 selected cities from 2003 to 2016. We observe that IEE shows an upward trend with fluctuations. Specifically, IEE increased slightly from 2003 to 2009, rose significantly from 2010 to 2013, and then gradually increased again after falling back in 2014. Environmental regulation intensity showed a clear upward trend, with minimal fluctuations. This suggests that during the study period, the Chinese government’s supervision on environmental issues grew rapidly, and the environmental regulation system was gradually established. The financial pressure curve showed an upward trend, with large fluctuations between 2007 and 2013. The Global Financial Crisis (GFC), which began in 2007, caused a sharp increase in financial pressure for China’s RBCs. After the end of the financial crisis in 2009, local financial pressure gradually decreased. From 2014 to 2016, the Chinese government further promoted fiscal and taxation reform. As a result, local government tax revenue declined, and financial pressure again began to increase.

**Figure 4:**
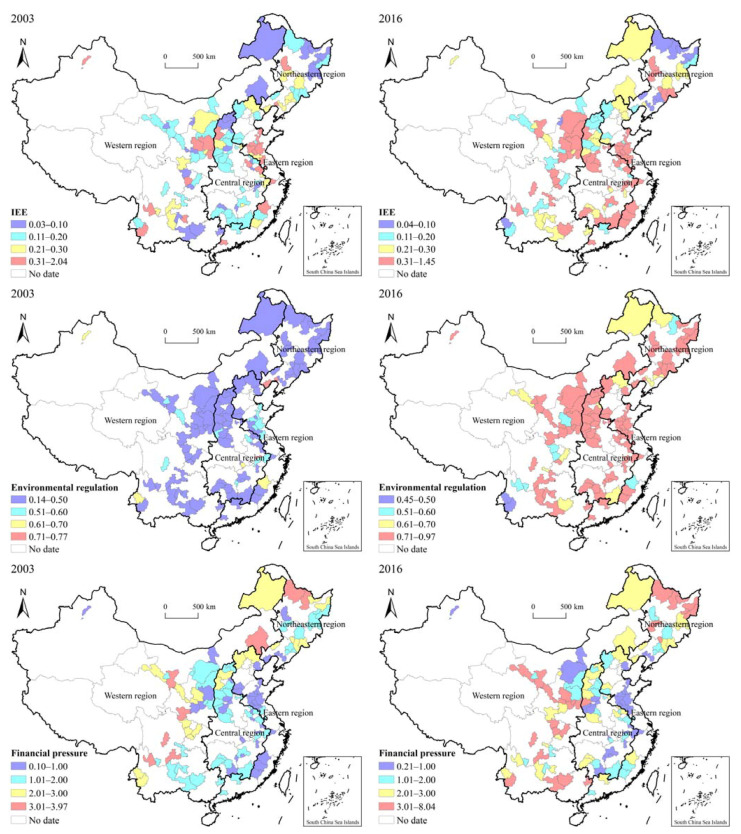
Spatial pattern of IEE, environmental regulation, and financial pressure in China’s RBCs.

Figure 4 shows the spatial pattern of IEE, environmental regulation intensity, and financial pressure in the subject cities in 2003 and 2016. Specifically, the areas with a high value of IEE expanded from the southeast coast to northwest inland areas, and IEE in the central and western regions improved significantly. This finding is consistent with the trend of industrial transfer in China since the beginning of the 21st century [69]. The change in the spatial pattern of environmental regulation intensity is clear. From 2003 to 2016, environmental regulation intensity in most cities increased from 0.5 to 0.7. Cities with less environmental regulation are scattered in the western and northeastern regions. The level of financial pressure was generally high in the western and northern regions and low in the eastern and southern regions. The financial pressure index was relatively high in the northeastern and western regions and is still increasing.

### 5.2. Regression Result Analysis

The Tobit regression model was used to test the effects of environmental regulation and financial pressure on the IEE of RBCs. The regression results are shown in Table 3. Models (1) and (2) are the regression models with environmental regulation and financial pressure, respectively, and model (3) is the regression model with the interaction term between the two.

We can observe from Table 3 that the coefficient of environmental regulation is significantly positive, indicating that tightening environmental regulation has a significant role in promoting the IEE of RBCs. With the tightening of environmental regulations, industrial enterprises accelerate the research and development of clean production technologies and promote improvement and innovation in products and processes to meet environmental protection standards and obtain stable profits. When innovation benefits offset or exceed regulatory costs and inputs, IEE effectively improves. Existing studies at different scales or on different regions reach the same conclusion [7,70]. Therefore, model (1) confirms Conjecture (1) that environmental regulation can promote IEE of RBCs in the theoretical analysis. The coefficient of financial pressure is significantly positive, indicating that local government financial pressure has a positive impact on improving IEE. Therefore, model (2) proves that Conjecture (2) that financial pressure will hinder the improvement of IEE in RBCs is not valid. This differs from the finding of Zhang et al. [32]. A possible reason for this is that, to relieve financial pressure, local governments may actively stimulate the vitality and endogenous power of industrial development. Moreover, governments vigorously support the development of key enterprises, revitalize industrial stock, improve industrial quality, reduce energy consumption, and thus improve efficiency. This promotes improvement in IEE to a certain extent. The coefficient of the interaction term between environmental regulation and financial pressure is significantly negative, meaning that when RBCs face the dual pressures of environmental regulation and financial difficulty, they may relax environmental regulation and introduce high-pollution and high-emission enterprises to alleviate financial pressure, which then inhibits improvement in IEE. Therefore, model (3) proves Conjecture (3) that the combined effect of environmental regulation and financial pressure will inhibit the improvement of IEE in RBCs. According to the analysis results in Figure 3, environmental regulation plays a decisive role in the impact of IEE. From 2003 to 2007, the intensity of environmental regulation and the level of financial pressure in RBCs were relatively low, and the IEE improved slowly, indicating that enterprises had poor environmental awareness at this stage, and the production process needed certain environmental regulation policies as constraints. From 2007 to 2009, the fiscal pressure increased obviously, but it did not bring about a substantial increase in IEE. It shows that the increase in local financial pressure will play a lesser role in promoting the improvement of IEE. After 2009, the intensity of environmental regulation increased while the financial pressure decreased, and the IEE showed a clear upward trend, indicating that, at this stage, the innovation revenue of enterprises was greater than the cost of environmental protection and innovation investment.

As for the control variables, industrial structure has a significant positive impact on IEE. As an important link between the economy and the environment, industrial structure plays an essential role in regulating resource consumption and pollution emissions [71]. After years of development and improvement, the industrial structure of RBCs in China has formed a virtuous circle with resources and the environment. The impact of economic development level on IEE is insignificant, indicating that its influence on IEE is unstable. This is consistent with Tan et al. [72]. Opening-up level has a significant negative impact on IEE. The reason for this is that when the opening-up level is high, many low-value-adding and high-polluting industries flood in, thereby increasing pressure on the local environment and resources and reducing regional ecological efficiency. This is consistent with the findings of Yang et al. [73]. Science and technology investment has a negative yet insignificant impact on IEE, indicating that the influence of science and technology investment on IEE is not clear. This finding differs from that of Chen et al. and Dai et al., both of which show that science and technology investment contribute to improved IEE [74,75]. In the long run, the impact of science and technology investment on ecological efficiency is complex and has a time lag. Therefore, the government should encourage enterprises to promote long-term investment in research and development so as to effectively improve IEE. The coefficient of industrial agglomeration is significantly positive, indicating that industrial agglomeration can improve IEE. This differs from the result of Gao et al. [76]. The reason for this lies in the external economy generated by the industrial agglomeration of RBCs. Geographic concentration of enterprises leads to deepening labor division, technology spillover, and intensive utilization of resources, which can in turn result in cost savings.

### 5.3. Heterogeneity Analysis

#### 5.3.1. Results Analysis for Different Economic Regions

The regression results for the four major economic regions (eastern, central, western, and northeastern) are shown in Table 4. Note from model (1) that environmental regulation has a significant positive impact on the IEE of cities in different economic regions. The eastern region has a long history of economic development, a solid economic foundation, and a high degree of industrial green development. Comprehensive environmental regulations prompt local enterprises to adopt advanced technologies to reduce costs. With the implementation of national strategies such as Western Development, Rise of Central China, and Reviving Northeastern Old Industrial Base, RBCs in the western, central, and northeastern regions have received a large amount of capital and technical support, which prompted enterprises to adopt green production processes and improve pollution treatment. Moreover, the promotion of ecological civilization construction improved the intensity of environmental regulation, which effectively inhibited the presence of polluting enterprises and improved IEE. Model (2) shows that the impact of financial pressure on IEE in the western region is significantly positive, suggesting that local governments have transformed financial pressure into fiscal incentives to expand tax sources. By seeking new fiscal revenue, economic growth in the western region is achieved, and at the same time, IEE is improved. The results for the eastern, central, and northeastern regions are insignificant. Furthermore, model (3) indicates that the interaction term between environmental regulation and financial pressure has a significant negative impact on cities in the eastern region and has no significant impact on cities in the central, western, and northeastern regions. When faced with the dual pressures of environmental regulation and financial difficulty, eastern cities tend to reduce environmental requirements and business access conditions to ensure economic development, which eventually inhibits improvements in IEE.

#### 5.3.2. Results Analysis for Different Economic Development Stages

The impact of environmental regulation and financial pressure on RBCs at different development stages shows clear heterogeneity (Table 5). From model (1), environmental regulation has a significant positive impact on the IEE of RBCs at different development stages. Since RBCs depend on the utilization of resources for a long time period, this inevitably leads to a series of ecological and environmental problems. The Chinese government has formulated a series of strict resource-intensive utilization and environmental protection policies to ensure the sustainable development of resource-based cities. This has to a large extent contributed to improvement in IEE. From model (2), note that the impact of financial pressure on mature cities is significantly positive, and the impacts on other cities are insignificant. The development of resources in mature cities is in a stable stage and such cities have strong economic resilience. When faced with financial pressure, they can adapt to the new economic environment by improving their technical level and adjusting the industrial structure. This has a positive role in improving IEE. Further, model (3) shows that the interaction between environmental regulation and financial pressure inhibits the IEE of growing and mature resource-based cities and promotes the IEE of recessionary and regenerative cities. Yet, only the coefficient of mature cities reaches statistical significance. When faced with financial pressure, growing and mature cities may reduce green aid to enterprises, relax environmental regulations, and ultimately inhibit improvements in IEE. Recessionary and regenerative resource-based cities are the key and pioneering areas for the transformation of the mode of economic development. When these two types of cities face financial pressure, they restrict entry of polluting enterprises through strict environmental regulations, and instead develop strategic emerging and modern service industries. By turning financial pressure into a driving force for development, IEE is greatly improved.

#### 5.3.3. Results Analysis for Different Resource Types

From the results of model (1) (Table 6), environmental regulation has a significant positive impact on the IEE of all cities. Under the concept of green development, a company’s existing production technology cannot maintain the original profit level. To comply with regulations and maximize profits, companies are forced to adjust their strategies and invest in technological innovation to improve IEE. From model (2), the impact of financial pressure on IEE of metal and non-metal resource-based cities is significantly positive. These two types of cities face serious resource depletion and high production costs. In response to financial pressure, local governments take effective measures to accelerate industrial transformation and upgrading. However, enterprises with high taxation attributes are introduced. In this way, financial pressure is transformed into a driving force for development, thereby improving IEE to a certain extent. The impact of financial pressure on cities with other resource types is insignificant. Note from model (3) that the interaction of environmental regulation and financial pressure has a significantly negative impact on metal resource-based cities, and a significantly positive impact on forest resource-based cities. The impact on other resource-based cities is insignificant. Metal resource-based cities are mainly in the Gansu, Shanxi, and Jiangxi provinces, which are currently in a critical period of industrial transformation and upgrade [77]. Environmental regulation and financial pressure pose great challenges for local enterprises, and the efficiency and benefits of such enterprises are affected, leading to a decrease in IEE. Since the Chinese government put forward the development concept of “clear waters and lush mountains are invaluable assets”, it has attached great importance to the development and protection of forest resources. When faced with the dual pressures of environmental regulation and financial difficulty, local governments adopt scientific development and utilization of forest resources, accelerate adjustment of the economic structure and the transformation and upgrading of the forest industry, and actively promote green innovation of economic activities, thereby improving IEE in forest resource-based cities.

### 5.4. Robustness Check

We used variable substitution to test the robustness of Tobit regression model [78]. That is, applied the CCR model (A data envelopment analysis model based on the assumption of constant returns to scale) to measure IEE and continue to use the Tobit model for estimate the established panel model again (Table 7). It can be seen that when the CCR model is used to measure the IEE, in the test results of all RBCs, although the absolute value and significance level have decreased, the sign and significance level have not changed. The regression results of different economic regions, different economic development stages and different resource types in the heterogeneity analysis are also basically consistent with the robustness test results in Table 7. In short, Tobit regression obtained robust and credible empirical results.

## 6. Conclusions

Based on data for 114 RBCs in China from 2003 to 2016, the Super-SBM model was implemented to analyze the IEE of these cities and their spatiotemporal characteristics. The Tobit model was used to analyze the mechanism of influence of environmental regulation and financial pressure on the IEE of RBCs. The main conclusions are as follows:

(1) Over the study period, the IEE of RBCs in China showed an upward trend with fluctuations. With continuous improvement in China’s environmental management policies, the intensity of environmental regulation has shown a clear increasing trend. The financial pressure of RBCs is greatly affected by both the economic situation and the reform of the fiscal and taxation system and fluctuates greatly.
(2) The IEE of RBCs in China shows a decreasing trend from the eastern region to the central region and on to the western region. The IEE of the northeastern and southwestern regions is still at a low level. The spatial heterogeneity of environmental regulation intensity is small. The level of financial pressure is high in the western and northern regions and low in the eastern and southern regions. The financial pressure in the northeastern and western regions remains in an uptrend.
(3) Appropriate environmental regulation and financial pressure have a positive impact on the IEE of RBCs. When faced with the dual pressures of environmental regulation and financial difficulty, improvement of IEE is inhibited. Industrial structure and level of industrial agglomeration can promote improvement of IEE, while the opening-up level has a significant negative impact on IEE. The impact of environmental regulation and financial pressure on cities in different regions, development stages, and of different resource types shows clear heterogeneity.

To improve IEE in RBCs, the government should fully utilize the positive externalities of industrial agglomeration, continuously optimize industrial structure, and establish a long-term effective mechanism for science and technology investment. Moreover, it is necessary to tighten the entry review of foreign investment-based projects and supervision of pollution treatment and adopt targeted and differentiated policies according to the characteristics of RBCs. From the perspective of economic regions, it is necessary to take advantage of the geographical advantages in the eastern region and actively learn from the useful experience of green industry manufacturing in developed countries. By strengthening environmental regulations, enterprises are urged to develop towards ecological optimization, thereby driving the progress of environmental protection technology in the central, western, and northeastern regions through tightening environmental regulations. In particular, eastern cities with high industrial ecological efficiency should provide key assistance to cities such as Heihe, Yichun, Hegang, and Baoshan where the level of IEE has always been low or has been declining. For areas with poor natural ecological endowments, the main driving forces for improving IEE are optimization of industrial structure and reasonable industrial agglomeration. From the perspective of the economic development stage, the financial budget system needs to be improved on the basis of appropriately strengthening environmental regulations, forming a constraint system that favors environmental protection, and expanding financial expenditure on environmental protection, especially in growing and recessionary cities. At the same time, we must pay attention to the construction of a low-consumption and high-efficiency industrial pattern, especially in terms of energy conservation and environmental protection, cleaner production, biomedicine, new energy and new materials, etc., to form scale advantages and technological advantages. We must develop large-scale, low-carbon industrial parks in qualified cities, optimize and adjust the layout of urban industrial parks, take industrial parks as the core, establish material and energy chains, create a circular economy, and develop clusters on a large scale. From the perspective of dominant resource type, cities need to actively learn from the development experience of other cities of the same type with high IEE and explore new industrial development models that are suitable for their own characteristics. Through preferential policies such as resource tax reduction and exemption, it is important to promote coal, metal, non-metal, and oil and gas resource-based cities to further promote supply-side structural reform, improve the adaptability and flexibility of supply structure to demand changes, and improve the market competitiveness of products. By increasing the intensity of environmental regulation, we must actively guide the industrial transformation and upgrading forest resource-based cities, improve total factor productivity, and promote green development.

## Figures and Tables

**Table 1:** Descriptive statistics of input and output variables.

Indicator	Category	Variable	Unit	Mean	Std. Dev.	Min	Max
Input	Environmental input	Industrial smoke and dust emissions	ton	44,016.49	178,979.98	139	5,168,812
		Industrial SO_2_ emissions	ton	65,332.57	56,291.14	612	337,164
		Industrial wastewater discharge	10 kilotons	4998.05	4357.63	122	29,365
	Resource input	Industrial electricity consumption	100 million KW·h	36.74	51.94	0.10	519.59
Output	Economic output	Industrial output	100 million Yuan	1282.89	1778.73	8.45	15,367.87

**Table 2:** Descriptive statistics of variables.

Variable	Indicator	Code	Mean	Std. Dev.	Min	Max
Dependent variable	Industrial ecological efficiency	*IEE*	0.285	0.331	0.126	3.617
Explanatory variable	Environmental regulation	*ER*	0.567	0.191	0.117	0.973
	Financial pressure	*FP*	1.836	1.724	0.002	17.399
Control variable	Industrial structure	*IS*	51.283	12.294	9.150	90.970
	Economic development level	*lnPGDP*	9.973	0.816	4.595	12.456
	Opening-up level	*FDI*	7.084	7.808	1.080	97.174
	Science and technology investment	*TEC*	0.843	0.967	0.201	20.683
	Industrial agglomeration	*IA*	2.299	3.228	0.008	20.988

**Table 3:** Regression results for all RBCs.

Variable	All Resource-Based Cities		
	(1)	(2)	(3)
*ER*	0.3461 ***		0.4010 ***
*FP*		0.0165 ***	0.0331 **
*ER*·*FP*			−0.0406 **
*IS*	0.0035 ***	0.0019 *	0.0037 ***
*lnPGDP*	0.0193	0.0925 ***	0.0221
*FDI*	−0.0034 ***	−0.0038 ***	−0.0034 ***
*TEC*	−0.0075	−0.0043	−0.0081
*IA*	0.0585 ***	0.0597 ***	0.0587 ***
*Constant*	−0.3881 ***	−0.8699 ***	−0.4773 ***

Notes: * *p* < 0.1, ** *p* < 0.05, *** *p* < 0.01.

**Table 4:** Regression results by different economic regions.

Variable	Eastern Region			Central Region			Western Region			Northeastern Region		
	(1)	(2)	(3)	(1)	(2)	(3)	(1)	(2)	(3)	(1)	(2)	(3)
*ER*	0.3410 **		0.8980 ***	0.3637 ***		0.1899 *	0.2934 ***		0.3077 **	0.3765 ***		0.2658 **
*FP*		0.0097	0.2903 ***		0.0001	−0.0797 **		0.0143 *	0.0194		0.0168	−0.0162
*ER*·*FP*			−0.5884 ***			0.1247			−0.0172			0.0477
*Control*	Yes	Yes	Yes	Yes	Yes	Yes	Yes	Yes	Yes	Yes	Yes	Yes

Notes: * *p* < 0.1, ** *p* < 0.05, *** *p* < 0.01.

**Table 5:** Regression results by different economic development stages.

Variable	Growing			Matured			Recessionary			Regenerative		
	(1)	(2)	(3)	(1)	(2)	(3)	(1)	(2)	(3)	(1)	(2)	(3)
*ER*	0.3224 ***		0.3382 ***	0.6156 ***		0.9844 ***	0.1909 **		0.1455	0.5010 **		0.3986 *
*FP*		0.0107	0.0097		0.0254 *	0.0080 ***		−0.0091	−0.0301		−0.0060	−0.0678
*ER*·*FP*			−0.0132			−0.1434 **			0.0316			0.0936
*Control*	Yes	Yes	Yes	Yes	Yes	Yes	Yes	Yes	Yes	Yes	Yes	Yes

Notes: * *p* < 0.1, ** *p* < 0.05, *** *p* < 0.01.

**Table 6:** Regression results by different resource types.

Variable	Coal-Based City			Metal-Based City				Nonmetallic-Based City			Forest-Based City			Oil and Gas-Based City		
	(1)	(2)	(3)	(1)	(2)	(3)		(1)	(2)	(3)	(1)	(2)	(3)	(1)	(2)	(3)
*ER*	0.1430 **		0.2334 ***	0.3779 ***		0.4535 ***		0.6366 ***		0.6833 ***	0.9667 **		−0.6262	0.6399 *		0.9808 **
*FP*		0.0032	0.0275		0.0234 ***	0.0477 ***			0.0244 **	0.0329		−0.0264	−0.3770 ***		0.0121	0.1604
*ER*·*FP*			−0.0485			−0.0469 **				−0.0433			−0.9184 ***			−0.2565
*Control*	Yes	Yes	Yes	Yes	Yes	Yes		Yes	Yes	Yes	Yes	Yes	Yes	Yes	Yes	Yes

Notes: * *p* < 0.1, ** *p* < 0.05, *** *p* < 0.01.

**Table 7:** Tobit regression test results.

Variable	All	Eastern Region	Central Region	Western Region	Northeastern Region	Growing	Matured	Recessionary	Regenerative	Coal-Based City	Metal-Based City	Nonmetallic-Based City	Forest-Based City	Oil and Gas-Based City
*ER*	0.2380 ***	0.3194 **	0.0296	0.1110	0.4215 ***	0.3662 **	0.2158 ***	0.0909 **	0.3389 **	0.1771 **	0.2914 ***	0.7532 ***	−0.2010	0.2548
*FP*	0.0071 *	0.0485	−0.0390	0.0043	−0.0138	0.0508 **	−0.0152	−0.0717	−0.0453	0.0212	−0.0268 *	−0.0018	−0.1011	0.0121
*ER·FP*	−0.0023 **	−0.1020 *	0.0791	0.0070	−0.0153	−0.0495	0.0204	−0.0771	0.0495	−0.0500 **	−0.0028	−0.0333	−0.1335 **	0.0006
*Control*	Yes	Yes	Yes	Yes	Yes	Yes	Yes	Yes	Yes	Yes	Yes	Yes	Yes	Yes

Notes: * *p* < 0.1, ** *p* < 0.05, *** *p* < 0.01.

## Data Availability

Not applicable.
